# The Origin of Cancer

**Published:** 1899-04-01

**Authors:** 


### THE ORIGIN OF CANCER.

Little by little we seem to be advancing towards the truth in regard to the real nature of cancer. Histological investigation has been fertile enough in suggestiveness, but has not gone further. Bodies have been found again and again in cancer cells, and it has been easy, perhaps too easy, to jump to the conclusion that these bodies were parasites. But proof has been wanting. A considerable step forwards, however, has recently been made. In a paper by Mr. H. G. Plimmer, read before a recent meeting of the Royal Society, certain investigations are described, the results of which point strongly to cancer being the result of an infection by a living organism. Nothing is more striking in cultivating micro-organisms than the peculiar susceptibility which many of them show to any variation in the medium in which they are being grown, and it is clear that an inability to cultivate a certain suspected organism often merely means an inability to discover the particular medium in which it will flourish. The medium used by Mr. Plimmer is an infusion made from cancer itself just as the ordinary beef infusion is made, to which a small proportion of glucose and tartaric acid has been added. When the air has been exhausted from this medium, so as to ensure anærobic conditions, and when it has been inoculated from selected specimens of cancer, a pure culture has been obtained of organisms similar to those observed in the original cancer cells. "These bodies correspond morphologically," says Mr. Plimmer, "with those found in the original tumour, and also with those described by Ruffer and myself, and by some others of those who have worked at the microscopical appearances of cancer." With the pure cultures so obtained several experiments have been made. Some have been without any result; but in some cases intraperitoneal injection was followed by the production of growths of an endothelial nature, "with the organisms within the cells and free in the tissues." Cultures made from these growths were in turn injected into fresh animals, and again the same sequence of events followed, growths developing containing the same organisms within their cells. Of course, these growths are not of an epithelial nature, but this may only be due to the fact that hitherto a suitable epithelium has not been found for inoculation.

The point of importance is that a malignant tumour has been produced in animals by an organism isolated from a malignant tumour in man. The deductions, then, which may be made from these experiments are: (1) That there are certain cancers in which there are, in enormous numbers, intracellular bodies of the kind described by Ruffer, Plimmer, and others as parasitic protozoa. (2) That by the use of appropriate means these intracellular bodies can be isolated and cultivated outside the body. (3) That these cultures, when introduced into certain animals, can cause death with the production of tumours, so far with one exception of endothelial origin, and that pure cultures can be made from these growths, which when inoculated will produce similar tumours. We need hardly say how great a step this is, nor need we say that its importance is all the greater in that its teaching seems to lie in the same direction as that of recent advances in operative surgery, in the direction, namely, of showing that cancer grows in response to a parasitic implantation, and that if the whole of the tissue in which the parasite has taken root can be removed the patient is once again in safety—at any rate, until a fresh implantation occurs. We must not forget, however, that although cancer may be shown to be parasitic, the fact that it grows once in a particular patient points to that patient being a fit medium for its developing afresh, unless, indeed, we are to believe that the doctrine of immunity may be found applicable even to cancer. Stranger things even than this are possible.

